# Genetic factors predict hybrid formation in the British flora

**DOI:** 10.1073/pnas.2220261120

**Published:** 2023-04-11

**Authors:** Max R. Brown, Peter M. Hollingsworth, Laura L. Forrest, Michelle L. Hart, Ilia J. Leitch, Laura Jones, Col Ford, Natasha de Vere, Alex D. Twyford

**Affiliations:** ^a^Institute of Ecology and Evolution, University of Edinburgh, Edinburgh EH9 3FL, United Kingdom; ^b^Tree of Life, Wellcome Trust Genome Campus, Hinxton CB10 1RQ, United Kingdom; ^c^Royal Botanical Garden Edinburgh, Edinburgh EH3 5LR, United Kingdom; ^d^Royal Botanic Gardens, Kew, Richmond, Surrey TW9 3AB, United Kingdom; ^e^Conservation and Research Department, National Botanic Garden of Wales, Llanarthne, Carmarthenshire SA32 8HG, United Kingdom; ^f^Spirent Communications, Positioning Technology, Crawley, West Sussex RH10 1BD, United Kingdom; ^g^Natural History Museum of Denmark, University of Copenhagen, DK-2100 Copenhagen, Denmark

**Keywords:** hybrid, floristic, genetic distance, DNA barcoding

## Abstract

Natural hybridization can have a profound evolutionary impact, with consequences ranging from the extinction of rare taxa to the origin of new species. Natural hybridization is particularly common in plants; however, our understanding of the general factors that promote or prevent hybridization is hampered by the highly variable outcomes in different lineages. Here, we quantify the influence of different predictors on hybrid formation across species from an entire flora. We combine estimates of hybridization with ecological attributes and a new species-level phylogeny for over 1,100 UK flowering plant species. Our results show that genetic factors, particularly parental genetic distance, as well as phylogenetic position and ploidy, are key determinants of hybrid formation, whereas many other factors such as range overlap and genus size explain much less variation in hybrid formation. Overall, intrinsic genetic factors shape the evolutionary and ecological consequences of natural hybridization across species in a flora.

## Significance

The importance of natural hybridization in the evolutionary process has intrigued biologists for decades. However, our general understanding of natural hybridization is impeded by the complex and often idiosyncratic outcomes of hybridization. Here, we investigate hybridization across all native flowering plant species in the intensely studied UK flora, combining floristic and ecological data with a species-level phylogeny. We show that parental genetic distance, phylogenetic position, and parental ploidy are the best predictors of hybrid formation, over and above many ecological factors such as parental range overlap. These results highlight the critical role of genetic factors in determining whether hybrids can form and establish in natural environments.

Natural hybridization plays an important role in evolution and may facilitate adaptation or the evolution of novel phenotypes and promote species survival, or alternatively, it can lead to a range of negative consequences such as a reduction in fitness and even extinction (1). Focused studies on evolutionary model systems have greatly contributed to our understanding of the role of natural hybridization in the evolutionary process, revealing how hybridization underlies the origin of new species (2–4) and may promote adaptation via introgression (5). These focused studies have selected species for their noteworthy hybrid outcomes, such as the recent formation of hybrid species (6) or large stable hybrid zones (7). However, it is currently unclear how findings from these individual studies apply more widely across the diversity of hybridizing species in natural communities. This is particularly the case in plants, where centuries of botanical exploration have revealed the complex and seemingly idiosyncratic outcomes of hybridization (8).

Gaining a general understanding of the factors that promote or prevent natural hybridization requires large-scale integrated analyses that include data for the diversity of species present in the wild. The few studies that have looked more broadly at hybridization in floras have revealed extensive variability in hybridity by plant group (9) and important associations such as between hybridity and perenniality (10, 11). However, previous work has largely considered hybridization and trait associations between genera and families and either lacks suitable phylogenies (11) or relies on those where the tips represent higher taxonomic units such as genera, families, or orders (9, 10). A species-level phylogeny is required to correct for the extensive phylogenetic nonindependence expected in many ecological traits as well as to infer how genetic distance and phylogenetic position affect hybridization.

In this study, we characterize the genetic landscape of natural hybridization across flowering plant (angiosperm) species in the United Kingdom. The UK flora is ideal for investigating natural hybridization as it is manageable in size for genetic characterization (~1,400 species are native or archaeophytes (12), i.e., introduced to the United Kingdom before 1500) but has sufficient diversity to include over 500 native hybrids and species representatives of ~20% of angiosperm families, allowing generalizations to be made based on comparisons between hundreds of hybridizing species pairs (12). There is also a hybrid flora—a unique resource describing the ecology, distribution, cytology, and parentage of natural interspecific hybrids (13, 14), i.e., hybrids that are sufficiently distinct from their parents and survive for long enough, or are produced frequently enough, to be recorded. Finally, there is a DNA barcoding resource—Barcode UK (15)—which includes the three-locus DNA barcode of *rbcL, matK*, and ITS2 for 1,479 native and archaeophyte UK flowering plant species. Here, we assess the determinants of hybridization across the flora, considering attributes of each genus (in terms of genus size and phylogenetic position) and differences between congeneric hybridizing species (range overlap, life history, ploidy, and genetic divergence), to investigate the general factors that determine the formation of hybrids across an entire flora.

## Results

Out of the 6,117 possible unique pairwise congeneric combinations between 1,100 species suitable for analysis (*Materials and Methods*), 480 (7.8%) are known to hybridize. From 244 genera containing multiple species, 96 contain reported hybrids (39.3%), while 148 (60.7%) do not (Dataset S1). These hybrids are disproportionately concentrated in just five genera from different plant orders, with 45.8% of hybrids found in *Euphrasia* (Orobanchaceae, Lamiales; n = 62), *Carex* (Cyperaceae, Poales; n = 50), *Rosa* (Rosaceae, Rosales; n = 40), *Epilobium* (Onagraceae, Myrtales; n = 35), and *Salix* (Salicaceae, Malpighiales; n = 33; Dataset S2). Some genera have multiple species that prolifically hybridize (e.g., *Euphrasia* has four species that are each parent to ≥14 hybrid combinations), while, in contrast, other genera have few species that overwhelmingly contribute to the number of hybrids (e.g., *Rumex crispus* is parent to 14 of ~40 *Rumex* hybrids). As such, hybridization is highly heterogeneous across the UK flora.

We next aimed to explain this heterogeneity in hybridization in two statistical models; model 1 included all factors except ploidy for 1,100 species, while model 2 additionally incorporated ploidy information and is limited to a subset of 684 species with ploidy data (*Materials and Methods*). We first consider potential nongenetic factors, including the number of species in a genus, species’ life histories, and parental species range overlap (hectad sharing).

Large genera have a greater number of potential parental combinations and thus may be predicted to include more hybrids (16). However, we find that the probability of congeneric species hybridizing is independent of genus size in our phylogenetic mixed models (pMCMC = 0.69 in model 1, *SI Appendix*, Table S1). Although hybridization tends to occur in species-rich genera, not all species-rich genera include hybrids (14). For example, clovers (*Trifolium*, 19 species) and lady’s mantles (*Alchemilla*, 12 species) are relatively species-rich but do not produce naturally occurring hybrids. This shows that the number of hybrid taxa deviates from a simple model determined by the number of congeneric taxa.

Previous studies at the genus or family level have shown annuality and perenniality to be drivers of hybridization over associated factors such as woodiness (11). We find that out of all potential congeneric species pairs in the British flora. Perennial–perennial (7.7%, n = 4,725) or annual–perennial parents (3.2%, n = 804) hybridize proportionally less frequently than annual–annual parental combinations (15.6%, n = 588). However, this pattern masks the disproportional effect of the large number of hybrids in the genus *Euphrasia*, and when removed, only 6.9% (n = 435) annual–annual combinations produce hybrids. Moreover, the overall higher abundance of perennial species in the flora results in 80% of hybrids being perennial (14). While life history effects on hybridization are complex and individual tests of life history prove nonsignificant in model 1 (pMCMC > 0.05; *SI Appendix*, Table S1), a Wald test of the joint difference in life history levels (annual–perennial and perennial–perennial parental pairs) accounting for phylogeny shows a significant effect on hybridization, with perennials more likely to hybridize (χ2 = 8.57, df = 2, *P* = 0.0138), confirming its importance.

The extent that parental species distributions overlap will affect the chance of cross-pollination and therefore hybrid formation (14, 17). We used underlying data from 40 million occurrence records to count parental range overlap in 10 × 10-km grids (hectads, see *Materials and Methods*). As expected, range overlap is higher for congeneric species pairs known to hybridize (739 hectads ±27 SE) than those that do not (353 hectads ±6 SE), and range overlap significantly affects the likelihood of hybridization (pMCMC < 0.001, *SI Appendix*, Table S1). Nevertheless, although significant, the variability in the effect of range overlap in the model is very low (posterior SD < 0.0001; *SI Appendix*, Table S1), especially compared to other factors (discussed below).

We next considered the genetic factors that may influence hybridization, in terms of parental ploidy, phylogenetic effects, and parental genetic distance and divergence time. Ploidy level variation is frequent in plant genera (17), with species with contrasting ploidy expected to have a lower likelihood of forming a hybrid due to endosperm imbalance in the fertilized embryo and potential meiotic abnormalities (18). For each pairwise congeneric comparison, we determined whether the parental species were of the same or different ploidy based on available flow cytometry (19) or chromosome count data (20) (Dataset S3). We find that species with the same ploidy are 35% more likely to produce hybrids than parents of differing ploidy, when fixed at a mean overlap in geographical distribution, mean branch length between species pairs, and accounting for phylogenetic effects (model 2, pMCMC < 0.001, *SI Appendix*, Table S2 and Figs. S1 and S2).

**Fig. 1.**
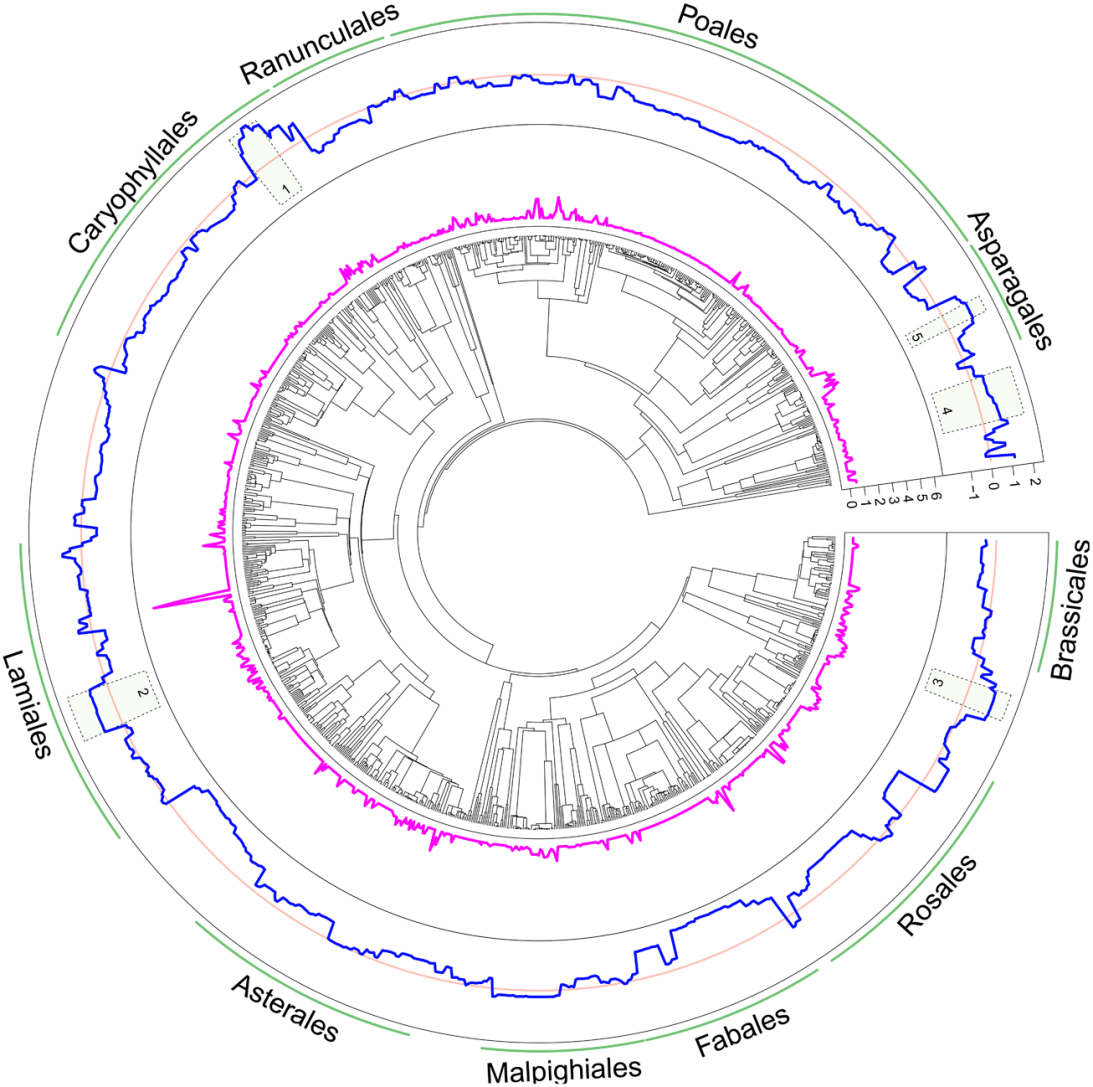
Distribution of hybrids across the phylogeny of the British flora. The innermost ring shows phylogenetic relationships of 1,100 British native flowering plant species based on a combined analysis of plastid DNA (*matK* and *rbcL*, alignment of all taxa) and nuclear ribosomal DNA (ITS2, gapped alignment for congeneric relationships only). Phylogenetic reconstructions used maximum likelihood implemented in IQ-TREE and were rendered into a time tree using treePL. The middle ring (pink line) shows the number of unique hybrid combinations a species produces weighted by genus size. The outer ring (blue line) shows the probit scale posterior mean of the probability of a particular species hybridizing. The zero line is represented in pale red with positive probit values indicating higher probabilities of hybridization. The figure is annotated with the five genera with the highest probabilities of hybridization, given variation in model fixed effects, indicated from the sum of the species-level posterior means from the MCMCglmm phylogenetic model [1. *Rumex* (Polygonaceae), 2. *Euphrasia* (Orobanchaceae), 3. *Epilobium* (Onagraceae), 4. *Potamogeton* (Potamogetonaceae), and 5. *Dactylorhiza* (Orchidaceae)], and the 10 largest plant orders around the outside.

We then investigated hybridization in the context of our 3-locus phylogeny (*Materials and Methods*). We find that the number of unique hybrid combinations a species produces weighted by genus size is highly uneven across the plant phylogeny (Fig. 1). The phylogenetic signal of hybridization in model 1 is high at 0.54 (0.31 to 0.69 CI—95% credible intervals; *SI Appendix*, Table S3), indicating that closely related lineages are likely to have more similar levels of hybridization. Inspection of the species-level best linear unbiased predictors (BLUPs; see *Materials and Methods*) from the phylogenetic model shows that docks and knotweeds (Polygonaceae) are most likely to hybridize after accounting for other model factors, while legumes (Fabaceae) are the least likely to do so (*SI Appendix*, Fig. S3).

**Fig. 2.**
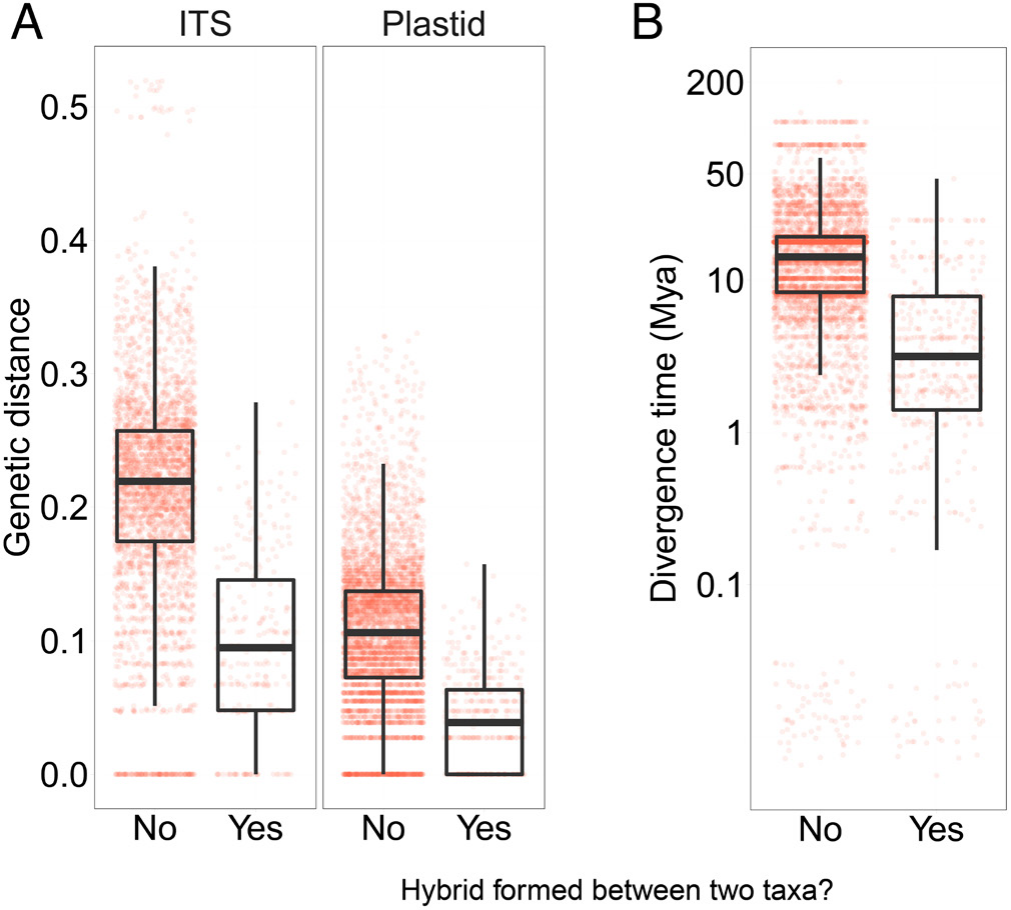
Hybrid formation in the UK flora in the context of (*A*) parental genetic divergence and (*B*) divergence time. Jittered points represent genetic distances between pairs of congeneric taxa, grouped by whether a pair of taxa produce a hybrid or not. Divergence times between taxa in panel *B* are from the dated phylogenetic tree combining ITS2 and plastid sequence data (*rbcL* and *matK*).

Finally, we considered the impact of parental genetic distance on hybridization. The likelihood of hybridization is expected to decrease with parental genetic distance due to increasing genetic incompatibilities as well as divergence in other traits (21). We observe a 10-fold variation in mean congeneric ITS2 distance (*Materials and Methods*) across the 35 genera that include more than five taxa and contain hybrids, from low mean pairwise distance in *Agrostis*, *Cochlearia*, and *Rosa* to high distances in *Geranium*, *Juncus*, and *Saxifraga* (Dataset S4). Overall, hybridizing congeneric species showed a significantly lower pairwise genetic distance (mean ITS2 distance = 0.097, SE = 0.004) and thus more recent divergence time in our time-calibrated phylogeny (4.45 Mya, SE = 0.63 Mya) than nonhybridizing congeneric species pairs (mean ITS2 distance = 0.215, SE = 0.001, 14.65 Mya, SE = 1.31; Wilcoxon test, *P* < 0.001; Fig. 2). A similar pattern is also observed with the plastid data (Fig. 2). Overall, the probability of hybridization strongly decreases as branch length between parental species increases in both statistical models (pMCMC < 0.001, *SI Appendix*, Table S1 and Figs. S4 and S5) and shows a greater standardized effect size than both pairwise range overlap (by five orders of magnitude, posterior SD: 3.77; *SI Appendix*, Figs. S1 and S2) and size of the genus.

## Discussion

Hybridization plays a profound role in natural ecosystems and is increasingly appreciated as an evolutionary stimulus across the tree of life (1, 22). However, it has proven a challenge to generalize across groups or predict likely outcomes due to the idiosyncratic findings of individual studies. Our work aims to understand the general predictors of hybridization across the diversity of plant species in an entire flora by using a standardized methodological approach. Using a species-level phylogeny and species traits for the flowering plants of the United Kingdom, we show the critical role of genetic factors such as parental genetic divergence in shaping the outcomes of hybridization, with our analyses also revealing the largely overlooked importance of cross-ploidy hybridization.

Genetic distance between parental species emerges as the strongest predictor of hybridization, with this variable known to predict the strength of reproductive isolation in laboratory-based experimental crosses (21, 23). In the UK flora, there are numerous genera characterized by low mean parental genetic distance, such as *Salicornia*, *Prunus*, *Rosa*, *Epipactis*, and *Atriplex*, with hybrids that form tending to be fertile (13). These contrast with rare examples of genera where hybrids form between divergent species, such as in *Saxifraga*, *Poa*, *Cardamine*, *Potamogeton*, and *Fumaria*, although the hybrids that do form tend to be sterile, suggesting limited opportunities for introgression (24). Overall, the difference in divergence age estimates between hybridizing and nonhybridizing taxa supports the pronounced reduction in hybrid fertility observed between species that diverged more than approximately 4 Mya (25). Causality in the link between hybrid formation and parental genetic divergence is not absolute, however, as low parental genetic divergence could also be a consequence of genetic homogenization following hybridization, which would reduce sequence variability. Recently diverged species within genera such as *Euphrasia* and *Salix* hybridize extensively, and plastid and nuclear ribosomal DNA introgression is likely (26, 27). Overall, these results show that parental genetic divergence is a good predictor of hybridization, though rare hybridization between divergent species may still have potentially important consequences, such as in polyploid hybrid speciation (28).

Our results also show the importance of other genetic factors in explaining the outcomes of hybridization. Differences in parental ploidy may result in reproductive barriers such as meiotic irregularities (18) and decrease the chances of hybridization, as we find in the UK flora (29). However, there are still 131 cross-ploidy hybrids (38% of hybridizing species pairs with ploidy information), and as such, ploidy can be considered a leaky rather than an absolute barrier to hybridization (30). Even if it occurs at a reduced frequency, cross-ploidy hybridization may have important evolutionary consequences, with ploidy differences partially sheltering the emergent hybrid lineage from homogenizing gene flow from at least one of the parents (5). We also report a high phylogenetic signal of hybridization, with the phylogenetic position being a good predictor of hybrid formation. This supports the findings of previous work based on mapping hybrid propensity on a phylogeny of plant orders (9). Variation in the frequency of hybridization by plant groups is well established; there are numerous examples of groups where hybridization is prevalent (e.g., orchids), while there are other cases such as the legume genera *Trifolium* and *Lathyrus* that have high species diversity but hybridize little (31). However, unpicking the underlying mechanisms of such phylogenetic effects is challenging, with variation potentially attributable to multiple traits of parental species, such as habitat preferences, chromosomal stability, and mating systems (32).

Other, nongenetic, factors have been reported to play an important role in the prezygotic isolation of plant species and, therefore, in hybridization (33). Factors such as genus size are expected to predict hybridization (9); however, we find no evidence of this. In the case of genus size, we attribute the lack of a clear-cut effect to the phylogenetic signal and genetic distance between species outweighing the increased opportunity for hybridization due to more congeners alone. Previous work has shown the importance of perennial life history in hybridization (10, 14, 34), at least in temperate floras (10), due to perennials being more highly outcrossing (35) and producing more gametes over a longer period of time (34). We detect complex effects of life history in the probability of hybridization, with the significance depending on the analysis. High recorder effort in the British Isles means that even ephemeral annual hybrids are routinely found (14), and this is particularly the case with the species-rich taxonomically complex annual genus *Euphrasia*. The genus is one of the few solely annual genera in the United Kingdom with many hybrids recorded (14, 36). However, life history is significant when we analyze hybridization across the flora accounting for phylogenetic effects, showing the importance of accounting for the nonindependence of life history across the phylogeny.

Hybridization is also constrained and shaped by the biogeography of parental species (37, 38). We find that hybrids are more likely to form when parental species distributions overlap more, which is likely due to increased opportunities for crossing events. However, this effect size is small, and our models suggest that broad-scale range overlap may not be a primary determinant of hybridization, with potential opportunities afforded by long-distance pollen dispersal, or range changes such as historical range overlap, confounding measures based on extant distribution ranges (14, 39, 40). There are also technical issues with such estimates, as parental species overlap remains a crude estimate due to the lack of resolution on fine-scale co-occurrence and does not take into account habitat change or levels of disturbance.

Overall, our study has highlighted how genetic factors have a profound impact on hybridization and shape the prevalence of hybrids across the British flora (13) and provides a useful comparative context for future large-scale integrated analyses of hybridization. Britain has a postglacial flora with high levels of disturbance, which is known to change the landscape of hybridization (41, 42); therefore, more studies are needed from other regions to understand the global landscape of natural hybridization.

## Materials and Methods

### Hybridization and Trait Data.

We extracted information on hybrids, their parental progenitors, and ploidy from the hybrid flora of the British Isles (13) and used the latest plant taxonomy according to the New Flora of the British Isles (12). The hybrid flora excludes the complex apomictic groups *Hieracium*, *Taraxacum*, and *Rubus fruticosus* agg. We further excluded a) hybrids known to have formed outside the British Isles (e.g., taxa introduced as hybrids), b) triple hybrids, c) dubious or doubtful hybrids, and d) crosses at below specific rank (subspecies, varieties). We also removed the rare cases of intergeneric hybridization (some Rosaceae, Poaceae, and Orchidaceae) due to model scaling issues associated with including all possible species combinations across the flora. The hybrid flora includes a single entry per hybrid combination; therefore, hybrids with multiple origins derived from the same parental taxa are counted only once, as in ref. 11. Downstream analyses led to further hybrids being excluded if there were no barcode data associated with the parental species, with this notably removing hybrids involving recently introduced species (i.e., neophytes, introduced post-1500).

Additional ploidy information was added from the Botanical Society of Britain and Ireland (BSBI) Cytology database (43) and the Kew Plant DNA C-values database (20) using custom Python scripts (see https://github.com/Euphrasiologist/web_mining). Species with multiple ploidy levels were excluded unless it was known which cytotype contributed to the hybrid. Life history (annual or perennial) of each species was extracted from the PLANTATT data on perennation (44), with the few biennials (<100) treated as perennials to reduce model complexity.

Pairwise range overlap (hectad sharing) was inferred using distributional data from the BSBI: https://database.bsbi.org.

### Phylogenetic Reconstruction.

We estimated phylogenetic relationships from the Barcode UK dataset (15), which includes a three-locus DNA barcode of *rbcL*, *matK*, and ITS2 for native flowering plant species. Complex or apomictic groups, as omitted from the hybrid flora (see above), were not sequenced (except for the manageable genus *Sorbus*, where suitable material was available). Due to the different sequence diversity and alignment success of plastid and nuclear ribosomal DNA across flowering plants, we used a single alignment of plastid sequences to infer relationships between all taxa, while nuclear ribosomal ITS2 was aligned separately for each genus and used to infer only congeneric relationships. Plastid DNA was aligned for all taxa using Geneious, while ITS2 was aligned by genus, padded with Ns, and gapped using the program catfasta2phyml (https://github.com/nylander/catfasta2phyml). Phylogenetic inferences were made using IQ-TREE (45) in an analysis with three partitions allowing models of molecular evolution to differ between loci and including a multifurcating constraint tree based on Angiosperm Phylogeny Group IV relationships (46) generated with Phylomatic (47). Tree support was estimated using 1,000 ultrafast bootstrap replicates (48). The phylogeny was dated using treePL, using calibration with 30 well-spread phylogenetically assigned fossils across the flowering plant phylogeny (49).

Tree-based genetic distances from the combined sequence alignment of ITS2 and plastid data were inferred using the R function cophenetic.phylo() from the package ape (50), while separate pairwise distances for ITS2 and plastid DNA were calculated with the R function dist.alignment() from the seqinr package (51). The resulting distances (either tree-based distances or pairwise distances) were the square root of pairwise distances. Tree manipulation took place in R, with the circular plot made with the R package circlize (52); the phylogeny was coerced into a circular dendrogram for visualization. Other plots were generated with the R package ggplot2 (53) and lattice (54). All other data manipulation took place in R version 3.6.1 using base R and packages data.table (55) and dplyr (56).

For visualization of the number of hybrids each species produces across the phylogeny, we summed the total number of unique hybrids for a given parental taxon and weighted this value by the size of the genus.

### Integrated Model-Based Analyses.

Our final dataset for the phylogenetic analysis contained 6,117 unique congeneric pairwise combinations, of which 480 produce hybrids. We used phylogenetic generalized linear mixed models implemented in the R package MCMCglmm as it allows for the addition of a phylogeny with flexible variance structures for the random effects (57). The response variable was a binary response of whether two congeneric species produced recorded hybrids or not and was assumed to have residuals approximated by a probit distribution (“threshold” model in MCMCglmm). We used parameter-expanded priors for better mixing and fixed the residual variance at 1. The models were run for 1.3 million iterations with a thinning interval of 1,000 and a burn-in of 300,000.

We used five fixed-effect covariates to understand their contribution to explaining the variation in hybrid formation: i) Pairwise branch length between parental species calculated from the phylogeny (above) was added to understand the contribution of intrageneric relatedness (=genetic distance); ii) pairwise overlap in geographical distribution (number of 10 × 10-km hectads (generated from data at https://database.bsbi.org/) accounted for the extent of range overlap of parental species (=hectad sharing); iii) genus size was calculated from species present in the phylogeny; iv) life history of the parental species combination was included as a three-leveled factor (annual–annual, annual–perennial, perennial–perennial) for each species combination in the model; v) finally, whether the parental species were of the same ploidy level or not was added as a two-level categorical factor: same ploidy level (homoploid) or not (heteroploid).

We ran two models which differed in their fixed effect structure only in terms of excluding or including ploidy data (model 1 and 2, respectively). This was because ploidy data were limited either due to missing or uncertain ploidy information; the number of species analyzed decreased from 1,100 to 684 when including ploidy level in the model. Out of 244 genera, 169 lacked ploidy information, with a few genera having high amounts of missing data, e.g., *Sorbus* and *Alchemilla*, as well as *Juncus* and *Carex*, which have holocentric chromosomes and whose ploidy level is therefore difficult to assess. The phylogenetic (species level) BLUPs (means of the posterior distribution of the model effects) were extracted from the model excluding ploidy level and are equivalent to the per species point estimates of the probability of hybridization.

The inverse relatedness matrix (unscaled phylogeny) and species were fitted as random effects in a multimembership model structure, as each hybrid event is the outcome of two parental species. The effect of phylogeny was added to the fixed effect predictions by calculating the following:

$$\sigma_{u}^{2}+2\sigma_{s}^{2}+\sigma_{p}^{2}\bar{B},$$

where *u* is the residual variance, *s* is the species variance, and *p* is the phylogenetic variance. $\bar{B}$ is defined as the average tree of all species pairs:

$$\bar{B}=2\bar{d}+2\bar{o},$$

where $\bar{d}$ is the average species phylogenetic variance, and $\bar{o}$ is the sum of the pairwise species phylogenetic covariances divided by the number of possible combinations of species multiplied by two. As we did not allow intergeneric hybridization, we calculated $\bar{B}$ at the species-level constrained to genus ( ${\bar{B}}_{S}$):

$${\bar{B}}_{S}=\frac{2\Sigma o_{i}nc_{i}+2\Sigma d_{i}n_{i}}{\Sigma {\mathrm{nc}}_{i}},$$

where i represents the $i^{\mathrm{th}}$ genus. Variables o and d are defined as above but calculated for each genus, $n_{i}$ is the number of individuals in a genus, and ${\mathrm{nc}}_{i}$ is the number of possible pairwise combinations within the genus. Using this method, we were able to account for the size of the genus in our phylogenetic variance estimates. We implemented this algorithm in the R package VCVglmm (58). Finally, the phylogenetic signal was calculated using:

$$\frac{\sigma_{p}^{2}{\bar{B}}_{S}}{\sigma_{u}^{2}+2\sigma_{s}^{2}+\sigma_{p}^{2}{\bar{B}}_{S}}$$

All parameters were sampled from the posterior distribution of each coefficient, and distributions were summarized using modes and highest posterior density intervals at the 95% level. *P*-values were taken directly from model output for continuous covariates or categorical covariates with only two levels, whereas for life history effects (i.e., annual or perennial status of parental species), Wald tests were used to jointly test all factor levels (59). *P*-values in this context are pMCMC values (58).

Genetic distance comparisons between different hybrids were made using Wilcoxon tests on the pairwise parental genetic distances, which do not assume that the distances approximate any distribution. The source code for all the analyses and figures can be found at https://github.com/Euphrasiologist/Floristic_DNA_Barcoding.

## Supplementary Material

Appendix 01 (PDF)

Dataset S01 (XLSX)

Dataset S02 (XLSX)

Dataset S03 (XLSX)

Dataset S04 (XLSX)

## Data Availability

Sequence alignments and phylogenies have been deposited in Dryad (https://doi.org/10.5061/dryad.9kd51c5np) (60).
