# Supplementary material for: Genetic factors predict hybrid formation in the British flora
Source: Proc Natl Acad Sci U S A. 2023 Apr 11;120(16):e2220261120. doi: 10.1073/pnas.2220261120 (PMC10120012; doi:10.1073/pnas.2220261120)
Supplement: Supplementary file 1 — Appendix 01 (PDF) [file pnas.2220261120.sapp.pdf]

Supporting Information for
Genetic factors predict hybrid formation in the British flora

Max R. Brown, Peter M. Hollingsworth, Laura L. Forrest, Michelle L. Hart, Ilia J. Leitch, Laura Jones, Col Ford, Natasha de Vere, Alex D. Twyford

Alex D. Twyford, Max R. Brown,
Email: Alex.Twyford@ed.ac.uk; mb39@sanger.ac.uk

This PDF file includes:

Figures S1 to S5
Tables S1 to S3

Other supporting materials for this manuscript include the following:

Datasets S1 to S4

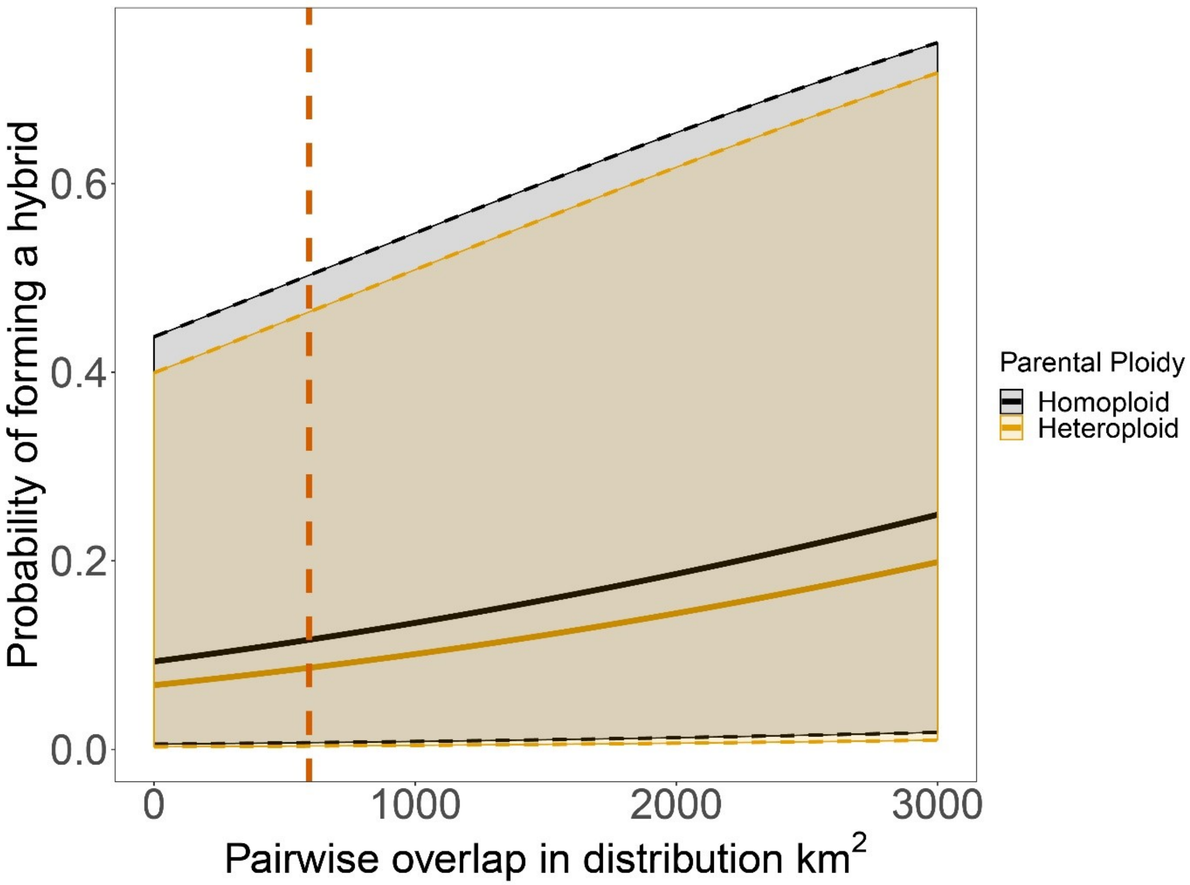

Fig. S1. Predicted fit of probability of hybridization given pairwise range overlap (hectad sharing) and ploidy levels of parental species from Model 2. Homoploid indicates parental species with the same ploidy level, and heteroploid indicates parental species with different ploidy levels. Dashed lines indicate the 95% Credible Intervals, and the bold lines represent the posterior mode of the coefficients of congeneric pairs of species hybridizing as a function of pairwise overlap in distribution, conditional on parental ploidy status. The effect is visualized at mean divergence time between all pairs of species for annual-perennial parent combinations and accounting for phylogenetic effects. The bold red dashed line indicates mean pairwise overlap in distribution (10x10km grids).

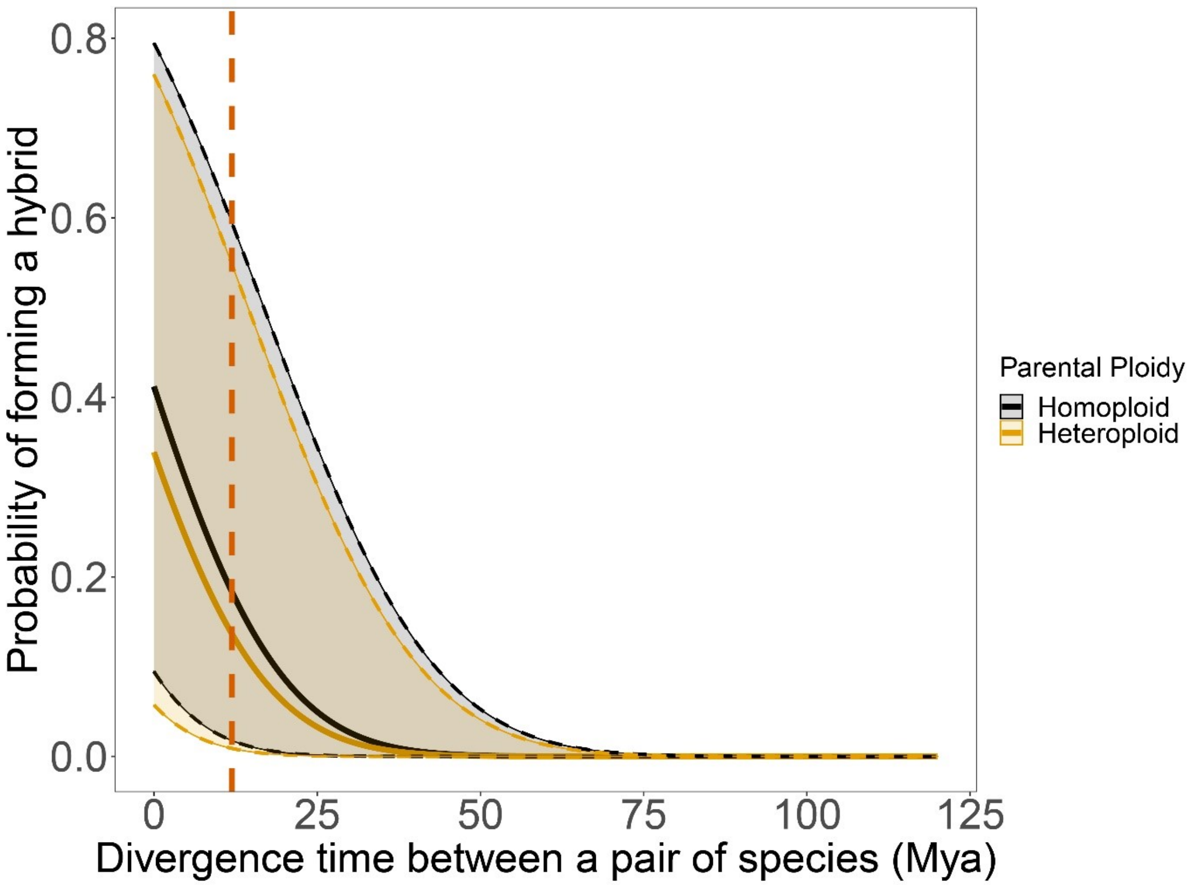

Fig. S2. Predicted fit of probability of hybridization given divergence time between parental species and ploidy difference of parental species from Model 2. Homoploid indicates parental species of the same ploidy level, and heteroploid indicates parental species of different ploidy levels. Dashed lines indicate the 95% Credible Intervals, and the bold lines represent the posterior mode of the coefficients of congeneric pairs of species hybridizing as a function of pairwise divergence time, conditional on parental ploidy status. The effect is visualized at mean hectad sharing for annual-perennial parent combinations and accounting for phylogenetic effects. The vertical red dashed line indicates mean pairwise divergence time between all pairs of species.

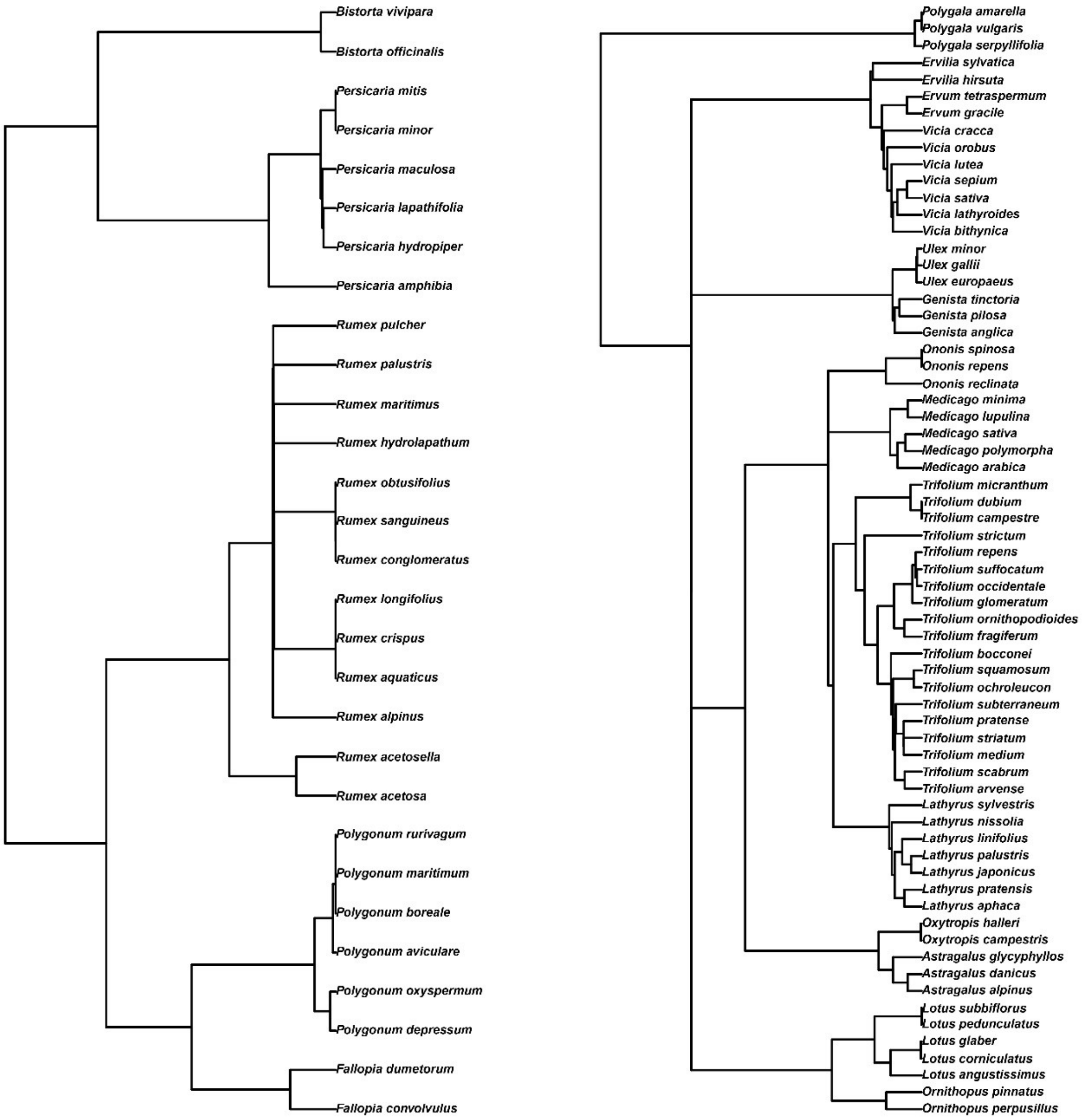

Fig. S3. Exemplar phylogenetic trees, for groups showing the highest and lowest posterior mean probability of hybridization in Model 1 (BLUP's of nodes in the phylogeny). The trees are extracted from our overall three locus flowering plant phylogeny for the UK flora. (A) shows the tree for the docks and knotweeds (subset of Polygonaceae) which are the group predicted to be most likely to hybridize, whilst (B) shows the tree for Fabaceae (with Polygalaceae as the outgroup), which is the family with the lowest probability of hybridization.

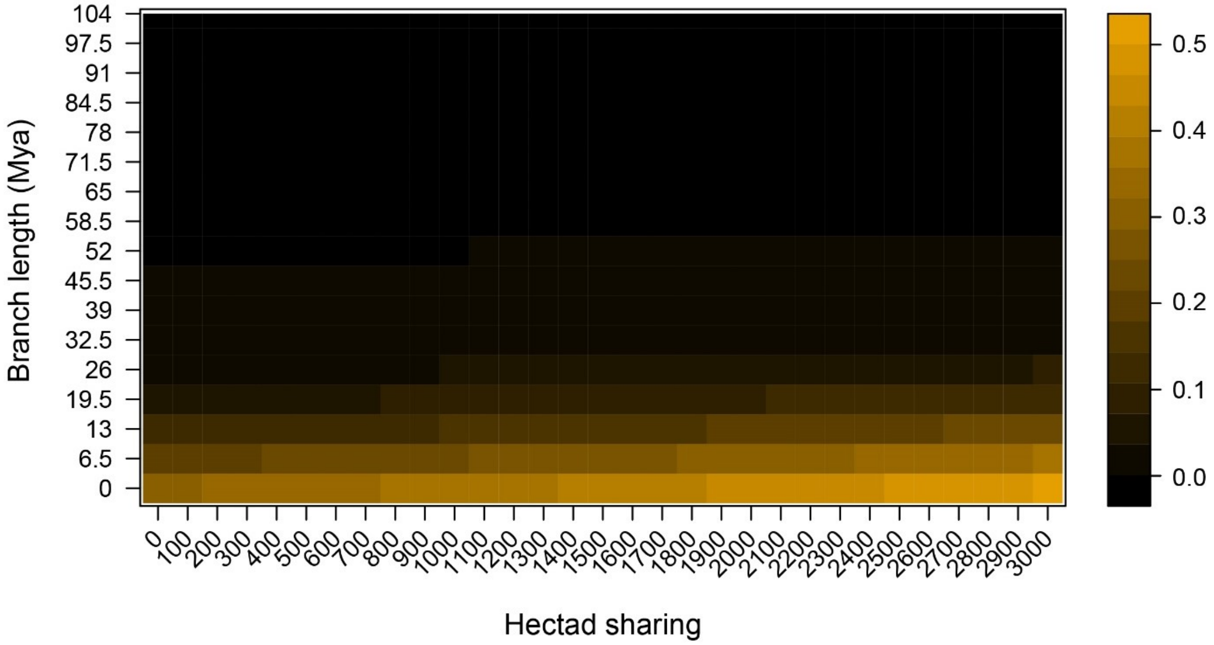

Fig. S4. The joint probability of hybridization between two parental species given both branch length between species (tree based divergence time measured in millions of years) and geographical overlap between parental species (measured as overlap in occupancy of 10x10km grid squares). The degree of shading in the scale bar and tiles represent the posterior probability of hybridization from Model 1 given parameter values for each variable. Estimates are visualized at mean genus size, for annual-perennial parental combinations and accounting for phylogenetic relationships between species.

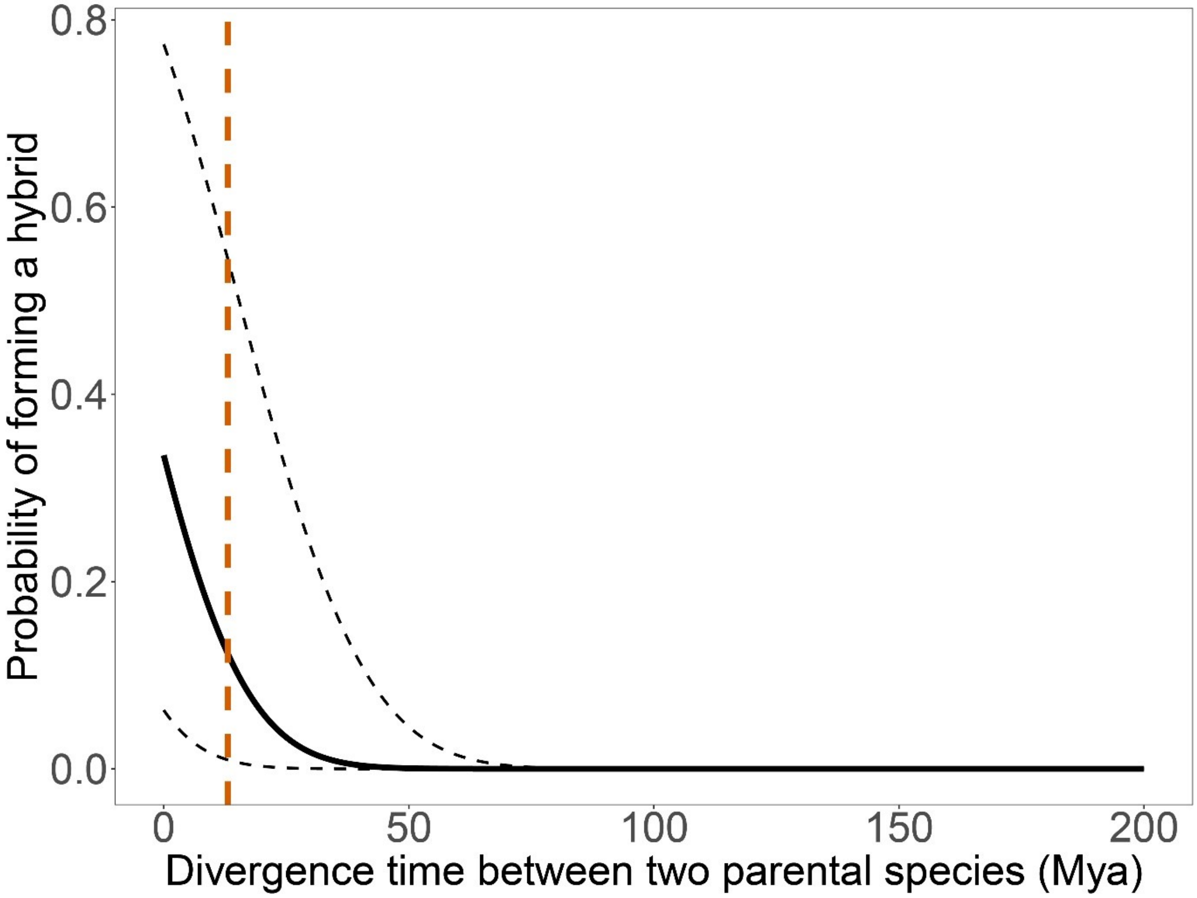

Fig. S5. Predicted fit of probability of hybridization given divergence time in millions of years ago (Mya) between parental species from Model 1. Black dashed lines are the 95% Credible Intervals, bold line is the posterior mean of the coefficient for the probability of congeneric pairs of species hybridizing as a function of branch length. This effect is visualized at mean hectad sharing, for annual-perennial parent combinations and accounting for phylogenetic effects. The bold red dashed line indicates mean genus level divergence time between pairs of species.

Table S1. Probability of hybridization with genus size, life history of parental species, range overlap (i.e. hectads shared) between parental species and parental genetic distance as fixed covariates (Model 1). The posterior mean of the distribution of each coefficient is given, along with lower and upper 95% Credible Intervals (CI). The p-value (pMCMC) is also reported and given in bold where significant. Posterior standard deviations of the posterior means are reported for this model.

	Posterior mean	l-95% CI	u-95% CI	Effective sample size	pMCMC	Posterior standard deviation (SD)
(Intercept)	-1.78	-3.71	0.32	1000	0.096	1.018
Branch length between species pairs (genetic distance)	-0.22	-0.24	-0.19	415	**0.0010**	0.013
Hectads shared between species pairs (range overlap)	0.0006	0.0004	0.0008	1000	**0.0010**	<0.0001
Annual-perennial parent pair	-0.44	-1.23	0.40	872	0.28	0.42*
Perennial-perennial parent pair	0.61	-0.41	1.51	1000	0.21	0.49*
Genus size	0.0081	-0.031	0.049	1000	0.69	0.020

Table S2. Probability of hybridization with ploidy, genetic distance, hectads shared and life history of parental species as covariates (Model 2). The posterior mean of the distribution of each coefficient is given, along with lower and upper 95% Credible Intervals. The p-value (pMCMC) is also reported and given in bold where significant.

	Posterior mean	l-95% CI	u-95% CI	Effective sample size	pMCMC
(Intercept)	-0.51	-2.52	1.64	1000	0.62
Branch length between species pairs (genetic distance)	-0.23	-0.26	-0.19	7134	<**0.001**
Cross ploidy effect	-0.76	-1.05	-0.47	1204	<**0.001**
Hectads shared between species pairs (range overlap)	0.0008	0.0006	0.0011	1000	<**0.001**
Annual-perennial parent pair	-0.60	-1.74	0.40	1000	0.28
Perennial-perennial parent pair	0.55	-0.55	1.84	1000	0.39
Genus size	-0.018	-0.080	0.036	1000	0.57

Table S3. Phylogenetic signal of probability of hybridization and the species variance independent of phylogenetic effects. 95% Credible Intervals of the variances are also presented. See methods for calculation.

**Variance Component**	**Posterior Mode**	**Lower Credible Interval**	**Upper Credible Interval**
Model 1 Phylogenetic Variance	0.54	0.31	0.69
Model 1 Species Variance	0.43	0.25	0.60
Model 2 Phylogenetic Variance	0.58	0.30	0.72
Model 2 Species Variance	0.43	0.13	0.45

Dataset S1 (separate file). Hybridization statistics for UK flowering plant genera. Data based on (1), with some groups excluded (see Materials and Methods).

Dataset S2 (separate file). Hybridization statistics for UK flowering plant species. Data based on (1), with some species excluded (see Materials and Methods).

Dataset S3 (separate file). Compiled ploidy statistics for UK hybridizing flowering plant species. Data based on (1) or (2); note some hybrids excluded (see Materials and Methods).

Dataset S4 (separate file). Genetic distance between species in hybridizing UK flowering plant genera. Mean pairwise genetic distance based on ITS2 sequences, for each of the 35 genera that include more than five taxa and contain hybrids.

SI References

1. C. A. Stace, C. D. Preston, D. A. Pearman, *Hybrid flora of the British Isles* (Botanical Society of Britain and Ireland, 2015).
2. J. Pellicer, I. J. Leitch, The Plant DNA C-values database (release 7.1): an updated online repository of plant genome size data for comparative studies. *New Phytologist* 226, 301-305 (2020).
